# Editorial: Diet, Nutrition, Supplements, and Integrative Oncology in Cancer Care

**DOI:** 10.3390/nu17213422

**Published:** 2025-10-31

**Authors:** Moshe Frenkel, Samuel E. Mathis

**Affiliations:** 1 Integrative Oncology Service, Oncology Division, Rambam Health Care Campus, Haifa 3109601, Israel; 2 Department of Family Medicine, The University of Texas Medical Branch at Galveston, Galveston, TX 77555, USA; semathis@utmb.edu

## Abstract

The Special Issue *“Diet, Nutrition, Supplements and Integrative Oncology in Cancer Care”* brings together emerging evidence and international perspectives on the pivotal role of nutrition and integrative approaches in oncology. Eight peer-reviewed articles explore the interface between dietary interventions, supplement use, and integrative care models. Collectively, these studies underscore that nutritional and complementary strategies are not peripheral but fundamental to improving patient outcomes, treatment tolerance, and quality of life. The collection has achieved wide dissemination—over 35,000 views and 33 citations—reflecting the growing global engagement with evidence-based integrative oncology. This Editorial summarizes the contributions of each paper, highlights cross-cutting insights, and situates their importance within a broader scientific and clinical context. Nutrition and dietary supplements are essential components of modern integrative oncology, supporting a transition toward patient-centered, holistic cancer care.

## Editorial

The integration of diet, nutrition, and evidence-based complementary modalities into cancer care has become one of the most dynamic and transformative trends in contemporary oncology. Historically, oncology has focused primarily on tumor-directed treatments—surgery, chemotherapy, radiotherapy, and targeted therapies—while other determinants of patient outcomes have received less systematic attention. However, as cancer survivorship grows and the biological complexity of the disease becomes more well understood, it is increasingly evident that optimal care extends far beyond cytotoxic or molecular interventions.

Malnutrition, sarcopenia, and metabolic dysregulation remain highly prevalent among patients with cancer, affecting 30–80% of individuals depending on disease site and stage. These factors are not merely secondary complications but powerful determinants of treatment response, toxicity, and survival. Evidence demonstrates that nutritional deficiencies correlate with higher rates of infection, prolonged hospitalization, decreased treatment adherence, and impaired recovery. Conversely, adequate nutritional support has been shown to improve functional status, mitigate side effects, and enhance resilience throughout the cancer continuum.

Diet and supplements thus serve dual functions: they sustain physiological integrity during therapy and contribute to a biological milieu less favorable to tumor progression. Beyond macronutrients, bioactive compounds—such as polyphenols, omega-3 fatty acids, vitamins, and plant polysaccharides—can modulate inflammation, oxidative stress, immune response, and metabolic signaling. These molecular pathways bridge nutrition science with oncologic outcomes and highlight the mechanistic foundation for integrative interventions.

Within this context, integrative oncology provides a conceptual and clinical framework for incorporating nutrition and supplementation into patient-centered care. Defined as the evidence-informed use of complementary therapies alongside conventional treatment, integrative oncology emphasizes the whole person rather than the tumor alone. Nutrition, physical activity, stress reduction, and psychosocial support together form a comprehensive therapeutic ecosystem that optimizes healing capacity and quality of life [1,2,3].

The Special Issue *“Diet, Nutrition, Supplements and Integrative Oncology in Cancer Care”* was established to advance scholarly discussion, showcase emerging evidence, and facilitate collaboration among clinicians and scientists working at this interface. To date, eight papers have been published, collectively representing a diverse and high-impact contribution to the field.

Akad et al. [4] compared total versus subtotal gastrectomy in gastric cancer patients and assessed postoperative nutritional and biochemical parameters. Their study demonstrated how surgical choice directly influences nutritional recovery and metabolic stability, emphasizing the centrality of nutritional follow-up and individualized diet planning in oncologic surgery.

Berretta et al. [5] presented data from a multicenter retrospective study evaluating an integrative medicine model for cancer care. Their findings reveal that combining evidence-based complementary therapies—including nutritional counseling and dietary supplements—with standard oncologic treatment can significantly improve patient-reported outcomes and quality of life. This real-world analysis exemplifies how integrative frameworks can enhance multidisciplinary coordination.

Keizman et al. [6] reported prospective phase II data on the use of modified citrus pectin in men with non-metastatic biochemically relapsed prostate cancer. The study demonstrated durable disease stabilization with minimal toxicity, suggesting that certain natural compounds may have biologically meaningful roles when studied systematically in well-defined patient populations.

Andreazzoli et al. [7] explored an integrative approach to multiple myeloma care, emphasizing nutrition, supplements, and complementary modalities as core elements of supportive management. The authors propose a conceptual model for multidisciplinary collaboration, aligning nutritional strategies with pharmacologic regimens to reduce toxicity and strengthen patient resilience.

Garutti et al. [8] offered a comprehensive review of nutritional management for oncological symptoms such as anorexia, cachexia, mucositis, and dysgeusia. They highlight early nutritional assessment and intervention as essential to mitigating side effects and improving functional outcomes. Their synthesis positions the dietitian as an integral member of the oncology team.

Frenkel et al. [9] proposed a structured, ethical framework for clinicians when patients request to add dietary supplements during treatment. The suggested model emphasizes open communication, shared decision-making, and critical evaluation of available evidence. This article directly addresses one of the most common and complex encounters in integrative oncology—how to balance patient autonomy with clinical responsibility.

Afonso et al. [10] conducted a systematic review of vitamin D supplementation in oncology, concluding that while mechanistic rationale and observational associations are strong, clinical evidence remains heterogeneous. They call for rigorously designed randomized trials to determine optimal dosing and target populations, illustrating the need for precision in supplement research.

Finally, Cevolani Pires et al. [11] examined omega-3 supplementation in patients with pancreatic neoplasms, showing favorable effects on nutritional status and inflammatory profiles. Their findings support the broader concept that nutritional modulation of inflammation can complement pharmacologic therapy in advanced malignancies.

Together, these eight papers illustrate the breadth of scientific inquiry at the intersection of diet, supplements, and cancer care. From mechanistic insights to clinical implementation, they collectively affirm that nutritional strategies are vital components of integrative oncology. Several overarching messages emerge.

First, nutrition and supplementation should be integrated early—beginning at diagnosis and extending through treatment, survivorship, and palliative care. Early intervention prevents deterioration of nutritional status and supports physical and psychological resilience. Second, evidence-based supplements, when appropriately selected and monitored, can complement conventional treatments without compromising safety or efficacy. Third, methodological rigor is paramount. As the demand for integrative approaches grows, only well-designed studies can provide the clarity needed for clinical guidelines and policy development.

The clinical relevance of these findings is underscored by converging international recommendations. The American Cancer Society emphasizes diet, physical activity, and weight management as central to both prevention and survivorship [1]. The joint SIO–ASCO clinical guidelines endorse integrative therapies—including nutritional counseling—as effective for symptom management during and after cancer treatment [2]. The World Cancer Research Fund and American Institute for Cancer Research likewise advocate for dietary patterns rich in plant-based foods and limited in processed meats, sugars, and alcohol as part of global cancer control strategies [3].

In integrative oncology, nutrition and supplementation are not adjunctive luxuries but evidence-supported necessities. They shape immune competence, metabolism, treatment tolerance, and emotional well-being. Moreover, nutrition provides patients with a sense of agency—a tangible way to participate actively in their own recovery. This empowerment is particularly significant in the face of chronic disease and uncertainty, aligning the biological and psychological dimensions of healing.

The impressive readership and citation data for this Special Issue—over 35,000 views and 33 citations—reflect a growing appetite for credible information in this space. Clinicians, dietitians, and researchers across disciplines are increasingly seeking practical frameworks to navigate the complex landscape of dietary claims and supplement use. By curating scientifically grounded content, this Special Issue contributes to elevating the discourse from anecdote to evidence.

Equally important is the diversity of its authorship. Contributors from Europe, the Middle East, and the Americas underscore the universal relevance of nutrition in oncology and the shared recognition that cultural and dietary diversity must inform care models. This international engagement fosters cross-pollination of ideas, supports regional adaptation of integrative practices, and promotes collaboration among institutions with complementary expertise.

As integrative oncology matures, several research priorities emerge. Future studies should explore the synergy between dietary interventions and conventional treatments, including immunotherapy and targeted agents. Advances in nutrigenomics and metabolomics hold promise for personalizing nutrition-based therapies, while longitudinal research is needed to clarify long-term survival and quality-of-life benefits. Health-policy analyses should assess the cost-effectiveness and accessibility of integrative nutritional services to ensure equitable implementation across healthcare systems.

Clinicians are encouraged to apply the lessons from these eight contributions in daily practice. Early referral to oncology nutrition specialists, systematic screening for supplement use, and open dialogue about evidence-based integrative options can transform the patient experience. Integrating diet and supplement discussions into multidisciplinary tumor boards and survivorship programs can also normalize their role within mainstream oncology.

The achievements of this Special Issue attest to the maturity and momentum of integrative oncology as a scientific discipline. They also reflect the dedication of authors, reviewers, and readers committed to advancing a holistic, evidence-driven approach to cancer care. By uniting research on diet, nutrition, and supplementation, this collection not only informs clinical practice but also inspires continued inquiry into how we nourish the body, mind, and spirit through the cancer journey.

In conclusion, diet and nutrition are not ancillary considerations but essential foundations of integrative oncology. Evidence-based supplementation, when applied judiciously, can further enhance the therapeutic landscape. Together, these interventions embody a vision of cancer care that is scientifically rigorous, humanistic, and patient-centered—a vision that this Special Issue both exemplifies and advances.

## References

[1] Rock C.L., Thomson C., Gansler T., Gapstur S.M., McCullough M.L., Patel A.V., Andrews K.S., Bandera E.V., Spees C.K., Robien K. (2020). American Cancer Society Guideline for Diet and Physical Activity for Cancer Prevention. CA Cancer J. Clin..

[2] Greenlee H., DuPont-Reyes M.J., Balneaves L.G., Carlson L.E., Cohen M.R., Deng G., Johnson J.A., Mumber M., Seely D., Zick S.M. (2017). Clinical Practice Guidelines on the Evidence-Based Use of Integrative Therapies During and After Breast Cancer Treatment. CA Cancer J. Clin..

[3] World Cancer Research Fund/American Institute for Cancer Research Diet, Nutrition, Physical Activity and Cancer: A Global Perspective. Continuous Update Project Expert Report 2018. https://www.wcrf.org/diet-activity-and-cancer.

[4] Akad F., Stan C.I., Zugun-Eloae F., Peiu S.N., Akad N., Crauciuc D.V., Moraru M.C., Popa C.G., Gavril L.C., Sufaru R.F. (2025). Nutritional and Biochemical Outcomes After Total Versus Subtotal Gastrectomy: Insights into Early Postoperative Prognosis. Nutrients.

[5] Berretta M., Quagliariello V., Ottaiano A., Santorsola M., Di Francia R., Carroccio P., Maurea N., Buonomo O.C., Facchini G., Di Mauro G. (2025). Multidisciplinary Integrative Medicine Approach for Cancer Patients: A Multicenter Retrospective Study. Nutrients.

[6] Keizman D., Frenkel M., Peer A., Kushnir I., Rosenbaum E., Sarid D., Leibovitch I., Mano R., Yossepowitch O., Margel D. (2023). Modified Citrus Pectin Treatment in Non-Metastatic Biochemically Relapsed Prostate Cancer: Long-Term Results of a Prospective Phase II Study. Nutrients.

[7] Andreazzoli F., Levy Yurkovski I., Ben-Arye E., Bonucci M. (2024). Conceptualizing an Integrative Multiple Myeloma Care: The Role of Nutrition, Supplements, and Complementary Modalities. Nutrients.

[8] Garutti M., Noto C., Pastò B., Cucciniello L., Alajmo M., Casirati A., Pedrazzoli P., Caccialanza R., Puglisi F. (2023). Nutritional Management of Oncological Symptoms: A Comprehensive Review. Nutrients.

[9] Frenkel M., Morse M.B., Narayanan S. (2023). Addressing Patient Requests to Add Dietary Supplements to Their Cancer Care—A Suggested Approach. Nutrients.

[10] Afonso M.L., Capelas M.L., Pimenta N.M., Santos T., Mäkitie A., Ganhão-Arranhado S., Trabulo C., Dias D.D.S., Neves P.M., Ravasco P. (2025). A Systematic Review of Vitamin D Supplementation in Oncology: Chance of Science or Effectiveness?. Nutrients.

[11] Cevolani Pires L.B., Salaroli L.B., Galvão de Podesta O.P., Haraguchi F.K., Lopes-Júnior L.C. (2024). Omega-3 Supplementation and Nutritional Status in Patients with Pancreatic Neoplasms: A Systematic Review. Nutrients.

